# Is the ≥1 cm Width of the Resection Margin in Benign and Borderline Phyllodes Tumor Necessary to Reduce Recurrence?

**DOI:** 10.1155/2024/1432313

**Published:** 2024-08-02

**Authors:** Nattakarn Changchit, Natthawadee Laokulrath, Pradit Rushatamukayanunt, Pongthep Pisarnturakit

**Affiliations:** ^1^ Division of Head Neck and Breast Surgery Department of Surgery Siriraj Hospital, Bangkok, Thailand; ^2^ Department of Pathology Siriraj Hospital, Bangkok, Thailand

## Abstract

**Background:**

Phyllodes tumors (PTs) are fibroepithelial neoplasms of the breast, with current treatment guidelines recommending wide excision to achieve surgical margins of ≥1 cm to minimize the recurrence risk. However, diagnostic challenges with core biopsy specimens often result in suboptimal surgical margins. This study aims to elucidate the correlation between margin status and PT recurrence, thereby informing surgical decision-making and enhancing patient outcomes.

**Methods:**

This single-center, retrospective study reviewed records of Thai women diagnosed with PTs between 2011 and 2018, collecting data on demographics, clinical presentation, surgical approach, tumor grade, size, and margin status. The primary endpoint was recurrence.

**Results:**

Among 165 PT cases analyzed—49.1% borderline, 38.2% benign, and 12.7% malignant—the overall recurrence rate was 13.9% (*n* = 23) over a median follow-up of 4.5 years. No significant difference in recurrence rates was observed between patients with negative resection margins <1 cm (ranging from <1 mm to 9 mm) compared to those with ≥1 cm (10.2% vs. 7.1%, *p*=1.00). Notably, in negative resection margins <1 cm group, a margin <1 mm (close margin) was associated with a significantly higher recurrence rate compared to margins of 1–9 mm (17.0% vs. 4.9%, *p*=0.04). Borderline PTs followed the overall trend, while benign PTs showed increased recurrence with positive margins. Multivariate analysis indicated a significant association between margins <1 mm and recurrence (adjusted HR = 10.78 (95% CI 1.32–88.07), *p*=0.027), highlighting an increased recurrence risk with more extensive positive margins.

**Conclusion:**

Our findings suggest that a wide surgical margin of ≥1 centimeter may not be necessary to prevent recurrence in benign and borderline PTs. Notably, surgical margins narrower than 1 millimeter substantially elevate the recurrence likelihood in cases of borderline PTs. Furthermore, the presence of positive surgical margins correlates with an increased recurrence rate in benign PTs. These findings highlight the critical need for a strategic approach in determining surgical margins, tailored specifically to the type of PT, to enhance patient outcomes effectively.

## 1. Introduction

Phyllodes tumors (PTs) represent rare fibroepithelial breast neoplasms, with a prevalence in Asian women notably higher at 3.8% of all breast neoplasms compared to 0.3–1% in European populations [1, 2]. WHO 2019 classified PTs into benign, borderline, and malignant types based on histological features [2], with benign and borderline tumors comprising the majority (62–74%, 18–25%) [3–5].

Recurrence rates in Asian women range around 12%, contrasting with 3–7% in Western populations, occurring typically between 14 and 36 months postresection [3–5, 9–12]. Margin status upon excision is considered the most important predictor of recurrence, especially for borderline and malignant tumors [2, 5, 9–11]. According to NCCN guidelines and previous studies, an adequate surgical margin of ≥1 cm width is considered an adequate margin for preventing the recurrence of PTs [8, 9].

In practice, diagnosing PTs poses challenges due to intratumoral stromal heterogeneity, leading to core biopsy's limited diagnostic yield of mild cellular part and high false-negative rates (32%) [6, 7]. When the diagnosis of fibroepithelial lesions is given in core biopsy specimens, most surgeons perform excision that leads to inadequate or positive margins at the first operation, necessitating subsequent wide excision or mastectomy.

However, the management of PTs is still controversial. Although a ≥1 cm width of the surgical margin is recommended, some retrospective studies and meta-analyses have reported that the surgical margin is not associated with recurrence. In addition, retrospective studies and meta-analyses found that the rate of recurrence was not lower in cases with a ≥1 cm width of the surgical margin [3–5, 12–15]. Tremblay-LeMay et al. also pointed out that a width of at least 1 mm might be an adequate margin to prevent recurrence [14].

To minimize unnecessary reoperations, this study primarily aims to investigate margin status and width as indicators of recurrence. It is hoped that our findings will provide additional data to support guidelines for PT management, which currently lack clarity and necessitate further investigation.

## 2. Materials and Methods

### 2.1. Study Design

This study is a single-center retrospective review of data from 2011 to 2018 that was approved by the Siriraj Institutional Review Board. Cases with a diagnosis of PTs without evidence of carcinoma in the final pathologic report were retrieved. The demographic data, clinical presentation, and surgical method were collected. Pathologic reports of both core biopsy and resection specimens, including resurgery specimens, were recorded, emphasizing tumor grade, size, and margin status. In our study, operational parameters for margin terminology were defined as follows: excision, denoting a procedure without specific margin considerations; wide excision, indicating surgical attention to achieve margins of at least 1 cm. We delineated “initial resurgery” as either wide excision or mastectomy performed subsequent to the initial excision, aiming to attain wider margins and mitigate recurrence. The margin status of all cases was reviewed by one pathologist and reclassified as follows:

(i) Positive margin [13] (Tumor touching ink)
  - Unifoci = 1 focal area of tumor at the margin and width ≤5 mm
  - Multifoci = 2 or more foci of tumor at the margin and each width ≤5 mm
  - Extensive foci = tumor present at the margin over a broad front and width >5 mm
(ii) Negative margin
  - <1 mm or close margin
  - 1–9 mm (Reported in millimeters)
  - ≥1 cm

Our primary outcome is a recurrent event that was diagnosed in the following surgical specimen after resection of the recurrent PTs. The patient outcome on follow-up (alive, dead of disease, and recurrence) was recorded. The duration of recurrence after surgery, location, and management after recurrence were also collected.

### 2.2. Statistical Analysis

PASW statistics version 18 (Mahidol Licensed Software) was used for all statistical analyses. The data were retrospectively collected from January 2011 to December 2018. Qualitative data are presented as frequency and percentages, presentation, BI-RADS, core needle biopsy results, type of breast and axillary surgery, initial resurgery, resection margin, and tumor grade. Quantitative data are reported as the mean and standard deviation for age, size, and resection margin and as the median and range for non-normal distribution for tumor size.

Comparisons of recurrence for overall and subgroup analyses of qualitative data, such as baseline characteristics and margins, were performed by the chi-square test or Fisher's exact test, and quantitative data, such as age, were analyzed by the two-independent sample *t*-test and Mann‒Whitney *U* test for tumor size. For recurrence time analysis, the Kaplan‒Meier method was determined to be significantly different from the log-rank test.

To determine factors associated with recurrence, Cox regression and backward elimination methods were performed after adjusting for confounding factors with a *p* value <0.2, and then the adjusted hazard ratio (95% Cl) was represented. A *p* value less than 0.05 was considered statistically significant.

## 3. Results

### 3.1. Patient Characteristics

A total of 165 patients with a diagnosis of PTs was recruited for our study. The median follow-up time was 4 years and 6 months. The median age of the patients was 48 years. The predominant preoperative feature of PTs was a palpable mass categorized as BI-RADS 4B. Core needle biopsy results indicated fibroepithelial neoplasms with increased stromal cellularity in the majority of cases (71.2%), while 10.1% were reported as fibroadenomas. The definitive diagnosis of PTs was conclusively established in only 10.1% of cases based on core biopsy specimens. Most patients underwent wide excision or excision without axillary lymph node biopsy or dissection. Approximately 20% of cases required initial re-excision procedures (Table 1).

### 3.2. Pathological Report

The composition of PTs in this study comprised 49.1% borderline, 38.2% benign, and 12.7% malignant tumor. The median tumor size was 5 cm (range 0.9–25 cm). We observed that the largest tumor size was associated with malignant type (*p* < 0.01); the median sizes of malignant, borderline, and benign tumors were 8, 6, and 4 cm, respectively.

Margin status analysis revealed negative margins (no tumor touching ink) in 82.4% of cases and positive margins in 17.6%. Among cases with negative resection margins <1 cm, they constituted 79.4%.

In 137 cases of negative resection margin <1 cm and positive margins, only 10 patients underwent initial resurgery. Within the initial resurgery cohort (*n* = 33), 91% achieved negative margins, with 76.7% having margins ≥1 cm. However, the positive margin remained 9% in this group (*n* = 3), and all patients underwent wide excision for reoperation.

Most patients without initial resurgery achieved a negative margin (80.3%), and the vast majority having negative resection margin <1 cm (95.3%). Positive margins were identified in 19.7% of cases (Table 2).

### 3.3. Recurrence

The overall recurrence rate in our cohort was 13.9% (*n* = 23), 15.9% in benign tumors, 11.2% in borderline tumors, and 19% in malignant tumors, with 21 cases of local recurrence (91.3%) and 2 cases of distant recurrence. Notably, all instances of local recurrence were confined to the same breast, with 76.2% recurring within the same quadrant. The median time to recurrence was 4 years.

**Figure 1:**
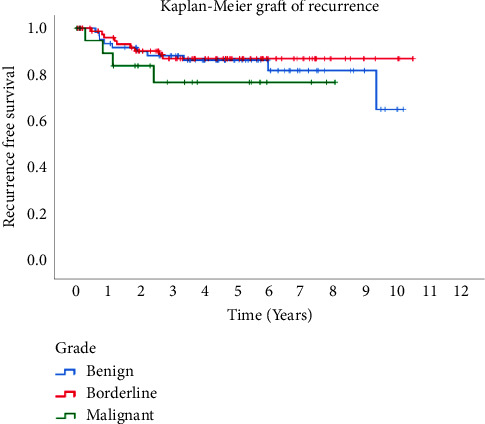
Recurrence-free survival of phyllodes tumors.

Factors including patient age, type of breast surgery, and axillary surgery did not exhibit significant associations with recurrence. Our study found no discernible relationship between the recurrence rate and the tumor type (*p* value = 0.48). Recurrence events occurred at median intervals of 3 and 6 years postsurgery in malignant and benign tumors, respectively (Figure 1).

### 3.4. Recurrence and Surgical Resection Margin

The width of the surgical resection margin is the most important predictive factor for PTs recurrence. *In the overall cohort*, which included benign, borderline, and malignant PTs, 34.5% of cases with positive margins experienced recurrence, which was a significantly higher rate compared to the 9.6% recurrence rate observed in cases with negative margins (*p* value = 0.002). *Within the cohort with negative resection margins*, widths of *<1 cm (ranging from <1 mm to 9 mm) or ≥ 1 cm did not demonstrate a significant association with recurrence* (<1 cm vs ≥1 cm; 10.2% vs 7.1%; *p* value = 1.00), a trend *consistent across both benign and borderline* PTs subgroups (Table 3).

*Within the negative resection margin <1 cm cohort* revealed a significantly *higher recurrence rate in cases with a resection width <1 mm* compared to wider margins (17.0% vs 4.9%; *p* value = 0.04). Furthermore, no recurrence was observed in the group with a negative surgical margin width >2 mm (Table 3).

*In benign tumors, recurrence rates exhibited a statistically significant increase exclusively among cases with positive surgical margins* compared to those with negative margins (40% vs 8%; *p*=0.009), while widths of negative margins <1 mm or wider did not demonstrate statistical significance in reducing recurrence (*p* value = 1, 0.086) (Table 3).

The results *within the borderline subgroup* mirrored the overall data, with cases featuring *positive margins and negative resection margins <1 mm displaying substantially higher recurrence rates* compared to those with negative margins ≥1 mm (30.80% vs 7.40%; *p*=0.030 and 16% vs 0%; *p*=0.032). Notably, negative resection margins >1 cm did not exhibit a statistically significant reduction in recurrence (9.1% vs 7%; *p*=1). Additionally, no recurrent events were observed during follow-up in cases with negative margins exceeding 1 mm in both benign and borderline tumors.

Due to the small sample size and limited events (*n* = 21) in the *malignant cohort*, a statistically significant difference in recurrence could not be detected.

In the positive surgical margin subgroup, there was no statistically significant difference observed in the overall recurrence rates among the unifocal, multifocal, and extensive positive margin subgroups, which stood at 25%, 33%, and 57.1%, respectively (*p*=0.33). Additionally, no significant discrepancy in recurrence rates was noted between the benign and borderline subgroups.

### 3.5. Subgroup Analysis excluding Mastectomy Cases

**Figure 2:**
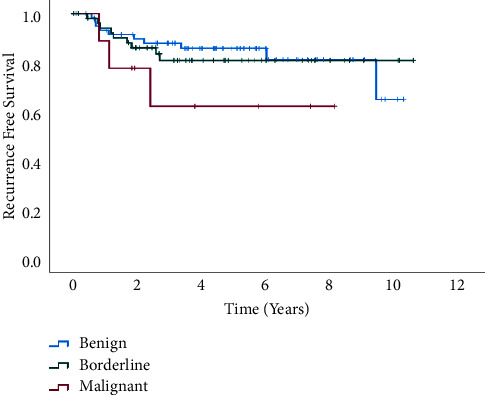
Recurrence-free survival of phyllodes tumors in subgroup analysis excluding mastectomy cases.

Subgroup analyses were conducted to explore potential modifiers of the association between resection margin status and recurrence rates in PTs. A total of 124 patients were included in the subgroup analysis, comprising those who underwent excision and wide excision procedures, while excluding cases of mastectomy. This subgroup encompassed *47.6% benign, 45.2% borderline, and 7.2% malignant PTs*. The median follow-up duration was 4 years and 6 months. Notably, *malignant cases exhibited the highest recurrence rate at 33.3%*, with a median time to recurrence of 2 years and 6 months. Conversely, *benign and borderline* cases demonstrated lower *recurrence rates at 16.9% and 16.1%*, respectively, with median recurrence times of 3 years and 6 months for borderline PTs and 4 years and 6 months for benign PTs (Figure 2). The observed *association between resection margin status and PTs recurrence was consistent with overall data analysis*. The status of surgical margins significantly impacts the likelihood of PTs' recurrence.

In the *benign cohort*, *only a positive resection margin demonstrated a statistically significant association with higher recurrence* rates compared to a negative margin (*p*=0.006), while a negative resection margin >1 mm did not yield a reduction in the recurrence risk.

Within *borderline PTs, recurrence rates were notably elevated in cases with margins <1 mm and positive margins* compared to those with margins of 1 mm or wider (*p*=0.017, 0.024) (Table 4).

However, owing to the limited sample size of the *malignant cohort* in the subgroup analysis (*n* = 9), an assessment of the relationship between margin status and recurrence in malignant PTs could not be conclusively determined.

### 3.6. Multivariate Analysis

In a multivariate analysis adjusted by grade, initial resurgery, and tumor size, the data showed a significant association between a <1 mm width, surgical margin, and recurrence. Furthermore, an increased risk of recurrence was identified in cases with more extensive positive surgical margins (Table 5).

## 4. Discussion

In this retrospective study, a meticulous examination of 165 cases of PTs was conducted, predominantly comprising benign and borderline subtypes. Core needle biopsy results revealed fibroepithelial neoplasms in the majority of cases (71.2%), while only approximately 10% of cases confirmed the diagnosis of PTs upon core biopsy analysis. This underscores the inherent challenge of accurately diagnosing PTs via core needle biopsy due to intratumoral stromal heterogeneity. Consequently, clinical correlation becomes imperative in the diagnosis of PTs, as their definitive exclusion cannot be based solely on core needle biopsy findings.

Due to the inherent difficulty in preoperative diagnosis, the management of surgical resection margins in each type of PTs presents significant challenges and remains a topic of controversy. Despite recommendations from the NCCN and Lu et al. [8, 9] advocating for a ≥1 cm width of the resection margin to prevent recurrence, our findings indicate that patients who underwent omitted resurgery experienced no recurrent events during a median follow-up of 4 years and 6 months. Within our institute, the management approach for patients with a <1 cm width of the surgical margin is tailored to individual patient characteristics and guided by clinical judgment, given the lack of definitive evidence supporting the adoption of wider resection margins and concerns regarding postoperative deformities in patients with smaller breasts.

Analysis of our series revealed a recurrence rate of 13.9% for PTs, exceeding reported data from Western countries but consistent with findings from Asian studies [3–5, 9–12] (Table 6). The median duration to recurrence was 4 years postsurgery. Among the three tumor types, malignant PTs exhibited the highest recurrence rate, with 33% of cases recurring within 2 years and 6 months postoperation, while recurrence rates for borderline and benign tumors were 16.9% and 16.1% at 3 years and 6 months and 4 years and 6 months, respectively. The majority of recurrent cases were localized within the same quadrant of the breast. Consequently, postoperative surveillance for recurrence must prioritize local recurrence and continue for at least 5 years.

The *most significant factor associated with PTs' recurrence is the surgical resection margin*, consistent with findings from other studies [2, 5, 9–11]. Our study meticulously examined the relationship between surgical resection margin and PTs' recurrence across overall data and subgroups defined by the tumor type, including subgroup analysis excluding mastectomy cases. Our findings consistently align with existing literature [3–5, 10–12, 15], *revealing no significant association between negative resection margins <1 cm or wider and recurrence risk in both benign and borderline PTs*.

*In benign PTs, only a positive resection margin demonstrated a statistically significant association with higher recurrence rates* compared to a negative margin (*p*=0.006), while a negative resection margin >1 mm did not reduce the risk of recurrence.

Conversely, *borderline PTs necessitate wider resection margins of more than 1 mm*, as both positive and negative resection margins <1 mm were strongly associated with higher recurrence rates in this subgroup (*p*=0.017). These findings align with those reported by Lim et al. [7], who stated that borderline PTs with a positive or less than 1 mm surgical margin have an increased risk of recurrence (Table 6).

Furthermore, extensive positive margins significantly influenced recurrence outcomes, as demonstrated in our multivariate analysis. Adjusting for grade, initial resurgery, and tumor size, our analysis revealed that both resection margins of less than 1 mm and more extensive positive margins were significantly associated with higher recurrence rates (<1 mm; adjusted HR = 10.78 (1.32–88.07); *p* value = 0.027).

Based on our findings, we propose a revised cutoff point for the appropriate surgical margin, *recommending a minimum width of 1 mm for borderline PTs and close margin for benign PTs to mitigate the risk of recurrence*. Given the limitations of preoperative biopsy in accurately identifying PTs, we suggest excising the tumor without rupture when clinical correlation indicates the presence of PTs and advocating for reoperation to achieve an adequate margin as delineated previously.

The strength of our study lies in the quantitative data collection of margin status (number of positive margins and margin width in millimeters) meticulously reviewed by a pathologist, enabling accurate recurrence analysis at each width. Additionally, our analyses consistently yielded congruent results across univariate and multivariate analyses and subgroup analyses excluding mastectomy cases.

Nevertheless, owing to the small sample size and limited number of recurrent cases from a single center, further investigations are warranted to corroborate our findings and reach definitive conclusions in malignant PTs.

## 5. Conclusion

In summary, while the magnitude of surgical resection margin plays a role in PTs' recurrence, a wide margin of ≥1 cm appears unnecessary for averting recurrence in benign and borderline PTs. Consequently, we posit a revised criterion for determining the optimal surgical margin. Specifically, we advocate for a minimum width of 1 mm for borderline PTs, coupled with close margin for benign PTs, thereby mitigating the risk of recurrence.

However, given the restricted dataset from a single center, additional research is warranted to validate these findings concerning malignant PTs.

## Figures and Tables

**Table 1:** Patient characteristic data.

	%
Age of diagnosis	
47.8 ± 10.83 years	
Presentation	
Mass	98.20
Abnormal screening	1.80
BI-RADS	
3	1.30
4A	32.30
4B	45.80
4C	11.60
5	9.00
Core needle biopsy	
Fibroepithelial neoplasm with increased stromal cellularity	71.20
Phyllodes tumor	10.10
Fibroadenoma	10.10
Spindle cell tumor	4.30
Others	4.30
Breast surgery	
Wide excision	39.40
Excision	35.80
Total mastectomy	24.80
Axillary surgery	
No	93.90
ALND	4.80
SLNB	1.20
Initial resurgery	
No	80.00
Yes	20.00
Re-wide excision	18.20
Total mastectomy	1.80

**Table 2:** Surgical resection margin data.

Margin	Overall		No initial resurgery		With initial resurgery	
	%	*n* = 165	%	*n* = 132	%	*n* = 33
Negative	82.40	136	80.30	106	91.00	30
Positive	17.60	29	19.70	26	9.00	3
Negative margin		136		106		30
≥1 cm	20.60	28	4.70	5	76.70	23
<1 cm	79.40	108	95.30	101	23.30	7
<1 mm	34.60	47				
Positive margin		29		26		3
Unifoci	55.20	16	53.80	14	66.70	2
Multifoci	20.40	6	19.20	5	33.30	1
Extensive foci	24.10	7	26.90	7	0.00	0

**Table 3:** Recurrence and margins in the overall, benign, and borderline cohorts.

Overall (*n* = 165)	Recurrent (%)	*p* value

Positive (*n* = 29)	34.50	0.002^*∗*^
Negative (*n* = 136)	9.60	
≥1 cm (*n* = 28)	7.10	1
<1 cm (*n* = 108)	10.20	
<1 mm (*n* = 47)	17.00	0.04^*∗*^
1–9 mm (*n* = 61)	4.90	

Benign (*n* = 63)	Recurrent (%)	*p* value

Positive (*n* = 15)	40.00	0.009^*∗*^
Negative (*n* = 48)	8.30	
≥1 cm (*n* = 13)	8.60	1
<1 cm (*n* = 35)	7.70	
<1 mm (*n* = 16)	18.80	0.086
1–9 mm (*n* = 19)	0.00	

Borderline (*n* = 81)	Recurrent (%)	*p* value

Positive (*n* = 13)	30.80	0.030^*∗*^
Negative (*n* = 68)	7.40	
≥1 cm (*n* = 11)	9.10	1
<1 cm (*n* = 57)	7.00	
<1 mm (*n* = 25)	16.00	0.032^*∗*^
1–9 mm (*n* = 32)	0.00	

^*∗*^Significant difference.

**Table 4:** Recurrence and margins in subgroup analysis with mastectomy exclusion group in benign and borderline cohorts.

Benign (*n* = 59)	Recurrent (%)	*p* value

Positive (*n* = 15)	40.00	0.006^*∗*^
Negative (*n* = 44)	9.10	
≥1 cm (*n* = 13)	9.70	0.836
<1 cm (*n* = 31)	7.70	
<1 mm (*n* = 16)	18.80	0.083
1–9 mm (*n* = 15)	0.00	

Borderline (*n* = 56)	Recurrent (%)	*p* value

Positive (*n* = 10)	40.00	0.024^*∗*^
Negative (*n* = 46)	10.90	
≥1 cm (*n* = 9)	11.10	0.980
<1 cm (*n* = 37)	10.80	
<1 mm (*n* = 16)	25.00	0.017^*∗*^
1–9 mm (*n* = 21)	0.00	

^*∗*^Significant difference.

**Table 5:** Multivariate analysis of recurrence and surgical margin.

Margin	Adjusted HR	95% CI lower	95% CI upper	*p* value
<1 mm	10.78	1.32	88.07	0.027^*∗*^
Unifocal positive	21.00	2.22	217.89	0.008^*∗*^
Multifocal positive	23.59	1.67	333.27	0.019^*∗*^
Extensive positive	28.44	2.15	376.35	0.011^*∗*^

^*∗*^Significant difference.

**Table 6:** Studies listing recurrence with attention to margin status.

	Recurrent rate					
	Changchit et al. 2024 [16]		Lim et al. 2021 [7]		Rosenberger et al. 2020 [3]	
	Thailand		Canada		USA	
Overall	Positive	34.5%	Positive	21.4%	Positive	2.7%
	Negative	9.6%				
	<1 mm	17.0%	<1 mm	10.0%		
					<2 mm	5.1%
	1–9 mm	4.9%	≥2 mm	2.0%	≥2 mm	2.0%

Benign	Positive	40.0%	Positive	16.7%	Positive	1.7%
	Negative	8.3%				
	<1 mm	18.8%	<1 mm	4.1%		
					<2 mm	2.7%
	1–9 mm	0.0%	≥2 mm	2.6%	≥2 mm	1.1%

Borderline	Positive	30.8%	Positive	50.0%	Positive	8.3%
	Negative	7.4%				
	<1 mm	16.0%	<1 mm	23.8%		
					<2 mm	12.5%
	1–9 mm	0.0%	≥2 mm	0.0%	≥2 mm	4.1%

## Data Availability

The data are included in the manuscript and available from the corresponding author upon request.
